# SNPs in IL4 and IFNG show no protective associations with human African trypanosomiasis in the Democratic Republic of the Congo: a case-control study

**DOI:** 10.12688/aasopenres.12999.1

**Published:** 2020-08-04

**Authors:** Olivier Fataki Asina, Harry Noyes, Bruno Bucheton, Hamidou Ilboudo, Annette MacLeod, Dieudonné Mumba Ngoyi, TrypanoGEN Group, as members of The H3Africa Consortium

**Affiliations:** 1 National Institute of Biomedical Research (INRB), Kinshasa, Democratic Republic of the Congo; 2 School of Medicine, University of Uele, Isiro, Democratic Republic of the Congo; 3 Center for Genomic Research, University of Liverpool, Liverpool, L69 7ZB, UK; 4 IRD-CIRAD 177, Montpellier, 34398, France; 5 Centre International de Recherche-Développement sur l'Elevage en zones Subhumides (CIRDES), Bobo-Dioulasso, Burkina Faso; 6 Institut de Recherche en Sciences de la santé ( IRSS)-Unite de Recherche Clinique de Nanoro( URCN), Nanoro, Burkina Faso; 7 Wellcome Centre for Molecular Parasitology, University of Glasgow, Glasgow, G12 8TA, UK; 8 School of Medicine, University of Kinshasa, Kinshasa, Democratic Republic of the Congo

**Keywords:** Human African Trypanosomiasis, T.b.gambiense, genes, polymorphisms

## Abstract

**Background: **Human African trypanosomiasis (HAT) is a protozoal disease transmitted by tsetse flies. Infection with trypanosomes can lead directly to active HAT or latent infection with no detectable parasites, which may progress to active HAT or to spontaneous self-cure. Genetic variation could explain these differences in the outcome of infection. To test this hypothesis, polymorphisms in 17 candidate genes were tested ( *APOL1 *[ *G1 and G2*], *CFH, HLA-A, HPR, HP, IL1B, IL12B, IL12RB1, IL10, IL4R, MIF, TNFA* *, IL6, IL4, IL8, IFNG*, and *HLA-G*).

**Methods: **Samples were collected in Democratic Republic of the Congo. 233 samples were genotyped: 100 active HAT cases, 33 from subjects with latent infections and 100 negative controls. Commercial service providers genotyped polymorphisms at 96 single nucleotide polymorphisms (SNPs) on 17 genes. Data were analyzed using Plink V1.9 software and R. Loci, with suggestive associations (uncorrected p < 0.05) validated using an additional 594 individuals, including 164 cases and 430 controls.

**Results:** After quality control, 87 SNPs remained in the analysis. Two SNPs in *IL4* and two in *IFNG* were suggestively associated (uncorrected p<0.05) with a differential risk of developing a *Trypanosoma brucei gambiense* infection in the Congolese population. The *IFNG* minor allele (rs2430561, rs2069718) SNPs were protective in comparison between latent infections and controls. Carriers of the rs2243258_T and rs2243279_A alleles of *IL4* and the rs2069728_T allele of *IFNG* had a reduced risk of developing illness or latent infection, respectively. None of these associations were significant after Bonferroni correction for multiple testing. A validation study using more samples was run to determine if the absence of significant association was due to lack of power.

**Conclusions:** This study showed no evidence of an association of HAT with *IL4* and *IFNG* SNPs or with *APOL1 G1 *and *G2* alleles, which have been found to be protective in other studies.

## Introduction

Human African trypanosomiasis (HAT), or sleeping sickness, is a neglected tropical disease caused by infection with extracellular blood protozoan parasites, which are transmitted by the bite of the tsetse fly ( *Glossina sp*) ( Headrick, 2014).

The disease is present in more than 250 foci in Africa and nearly 70 million people are at risk of infection with the two human-infective forms. A chronic form is found in Central and Western Africa and is caused by *Trypanosoma brucei gambiense* ( *Tbg*); an acute form is caused by *Trypanosoma brucei rhodesiense (Tbr*) and is found in East Africa. About 97% of all HAT cases are due to *Tbg* infection, which causes a chronic disease with a long latency period ( Kennedy, 2013).

There is currently no vaccine against HAT. The control of *Tbg* HAT is mainly based on the active detection of infected cases followed by their treatment and also vector control. After infection, the parasites may be undetectable by microscopy for months or years; these cases are known as latent infections and are detected by serology. Active HAT with microscopically detectable parasites evolves in two successive stages: stage 1 (haemolymphatic) and stage 2 (meningoencephalitis, also known as neurological). The transition from stage 1 to stage 2 is a consequence of the parasites crossing the blood-brain barrier. For a long time, HAT was considered fatal if untreated; however, the dogma that HAT is 100% fatal has been questioned ( Jamonneau *et al.*, 2012). It is now recognised that some individuals self-cure after developing a latent infection.

The Democratic Republic of the Congo (DRC) is the country with the highest incidence of HAT. More than half of districts are endemic for HAT, with more than 36 million people at risk ( Report of a WHO Expert Committee, 2013). There was an increase in the number of new cases at the end of the 20th century in both rural and urban areas; however, the number of cases is currently decreasing, with fewer than 3,000 cases detected in 2015 ( Büscher *et al.*, 2017).

There are several HAT foci in DRC with very different clinical presentations and outcomes, which could be explained by the presence of genetically different subpopulations of humans and possibly parasites, as well as environmental factors ( Kande Betu Kumesu, 2012). There is also variation in disease phenotype within populations, which may be partly due to individual genetic variation.

Several genes have been implicated in the control of other infectious diseases (AIDS, tuberculosis, malaria, etc.), but genetic data on HAT are more recent and incomplete. For example, differences in cytokine levels and other immunological markers may be a consequence of differences in disease states or of genetic differences between individuals in these cytokine genes or in genes that regulate them.

Polymorphisms in *IL6, IL10, IL1A* and *TNFA* have been associated with risk of developing HAT ( Bucheton *et al.*, 2011; Courtin *et al.*, 2006; Courtin *et al.*, 2007) in the DRC, although only the association with *IL6* remained significant after Bonferroni correction. An *HLA-G* haplotype has also been associated with HAT susceptibility in a Congolese population ( Courtin *et al.*, 2013).

It has also been reported that levels of interleukins *IL8* and *IL10* increase during infection and then decrease after treatment ( Lejon *et al.*, 2002) and that the stages of the disease are also correlated with levels of *IL6* and *IL10* ( Ilboudo *et al.*, 2012; Sternberg *et al.*, 2005). Our study is designed to investigate polymorphisms in candidate genes that have previously been implicated in the outcome of HAT, in an active focus in DRC.

## Methods

### Ethical statement

The study protocol was submitted to the ethics committee of the National Ministry of Public Health (DRC) and approved under number 1/2013 and by the Ethics Advisory Committee (EACC) of the Institute for Research and Development (IRD), Marseille.

All enrolled subjects signed an informed consent form written in French which was translated into the local language if necessary. Parents or guardians signed consent forms on behalf of minors when they couldn’t sign themselves and they had given verbal consent.

### Study design

This study was one of six studies of populations of HAT endemic areas in Guinea, Cameroon, Ivory Coast, DRC, Malawi and Uganda ( Ahouty *et al.*, 2017; Kabore *et al.*, 2017; Kamoto *et al.*, 2019; Kimuda *et al.*, 2018; Ofon *et al.*, 2017). The studies were designed to have 80% power to detect odds ratios (OR) >2 for loci with disease allele frequencies of 0.15 – 0.65, with the 96 single nucleotide polymorphisms (SNPs) genotyped for 100 cases and 100 controls.

### Study site

Our study was conducted in the district of Masi-Manimba, in the province of Kwilu, which was formerly a part of Bandundu province, in the south-west of the DRC, more than 388 km from Kinshasa.

### Recruitment

The study was undertaken in two stages: 1) a screen of 96 candidate loci in 233 subjects and 2) a validation study of two loci that were suggestively positive in the first round in 594 additional subjects.

Subjects were aged at least 12 years old and were enrolled in this study from July 2013 to March 2016 during the active screening campaigns of the mobile team of the National Program of Control of Human African Trypanosomiasis (PNLTHA). Participants were recruited in their respective villages during active screening campaigns. Simultaneous serological (Card Agglutination Test for Trypanosomiasis; CATT) testing and parasitological examination were performed. Samples that were CATT positive and lymph node fluid or Buffy coat positive for trypanosomes by microscopy were subject to the immune trypanolysis test, which is highly speciﬁc for *T. b. gambiense* ( Van Meirvenne *et al.*, 1995).

The immune trypanolysis test allows to confirm or not the presence of *T. b. gambiense* specific antibodies in an individual. Individual's plasma was collected in a 2 ml cryotube in the field, kept in a cryocontainer filled with liquid nitrogen and brought to the laboratory where plasma was stored in deep freezer -80°c. Live particular strains of *T. b. gambiense*, LiTat 1.3 and LiTat 1.5, were incubated with plasma as well as with guinea pig serum, serving as a source of complement. If the *T. b. gambiense* specific antibodies are present in the plasma, they bind to trypanosomes that will be lysed. Absence of trypanosome mobility, observed under a microscope, implied a positive result.

Cases (stages 1 and 2) were recruited from the positive test subjects for CATT, for whom parasitological evaluation confirmed the presence of the parasites in blood or lymph node fluid (for stage 1) and/or cerebrospinal fluid (CSF) (for stage 2).

Controls were CATT negative individuals living in the same area as cases and for whom the trypanolysis test was negative.

The subjects with latent infections were individuals who were negative by microscopy despite positive CATT and positive trypanolysis test results.

Controls and subjects with latent infections were enrolled as a comparison group. The first have never been in contact with *T.b.gambiense* , the latter have been in contact with *T.b.gambiense* but in this group, the parasite is no longer found in the absence of any treatment.

Individuals who could not provide a sufficient volume of blood (less than 9 ml) or were under 12 years old were excluded. Previously treated patients were not included as controls.

### Sample collection and processing

At inclusion, blood was collected through venepuncture by a skilled technician of PNLTHA using two heparin tubes of 5 ml. The first tube was used for serological (CATT) and parasitological examinations (capillary tube centrifugation [CTC], or mini-Anion Exchange Column Trypanosoma on Buffy Coat [mAECT-BC]). The second tube was used to collect plasma and buffy coat in 2 ml cryotubes. These two last samples were taken after centrifugation at 3500 rpm of the blood in the heparinized tube for 5 minutes. In addition, if the individual had shown enlargement of cervical ganglia, they were punctured and lymph node fluid examined by direct examination for trypanosomes. Once the trypanosome was isolated either in lymph node fluid or in the blood, the cerebrospinal fluid (CSF) was collected by lumbar puncture and examined for stage determination of the disease according to PNLTHA instructions. Plasma and Buffy coat were kept in containers filled with liquid nitrogen until they were sent to INRB where plasma was stored in the freezer at -20 ° C and Buffy coat in liquid nitrogen for subsequent DNA extraction for genotyping. DNA was extracted from Buffy Coat using the Maxwell16 Promega kit following the manufacturer's instructions (Maxwell®16 Tissue DNA Purification kit, cat# AS1030), quantified by Nanodrop (Thermo Scientific Nanodrop 2000, ISOGEN, Life Science) and then stored at -20°C. All DNA were sent to Makerere University, Uganda where they were quantified by Qubit ^®^3.0 Fluorometer (Invitrogen by Life Technologies) prior to shipment for genotyping. DNAs were shipped at room temperature. Transportation lasted less than 48 hours.

### Genotyping

Samples were submitted to Plateforme Genome Transcriptome de Bordeaux at INRA Site de Pierroton. A multiplex assay (two sets of 40 SNPs) was designed using Assay Design Suite v2.0 (Agena Biosciences). SNP genotyping was achieved with the MassArrayiPLEX genotyping assay using the iPLEX Gold genotyping kit (Agena Biosciences, cat# 10148-2) described in Gabriel *et al.* (2009). Products were detected on a MassArray mass spectrophotometer and data were acquired in real time with MassArray RT software 4.0.0.2 (Agena Biosciences). SNP clustering and validation was carried out with Typer 4.0.26.75 software (Agena Biosciences). All monomorphic SNPs and loci displaying more than three clusters of genotypes or unclear cluster delimitation were filtered out. 11 SNP that failed genotyping on the MassArray platform and 16 additional SNPs were genotyped by LGC Genomics, Hoddesden, UK (rs1143629, rs3212227, rs2546890, rs1130363, rs1233330, rs1818879, rs1801275, rs1424241, rs8062041, rs7185840, rs152828, rs375947, rs11575934, rs11548056., rs136174, rs73885316, rs136177, rs143830837, rs71785313, rs1136754, rs1059563, rs1136903, rs1136749, rs2021171) using the PCR-based KASP assay ( Semagn *et al.*, 2014), as were the SNPs tested in the validation study.

### SNP selection

SNPs were selected in 17 genes using two strategies. First, SNPs that were previously reported to have associations with HAT or other infectious diseases were selected in *APOL1* ( *G1* and *G2* SNPs), *CFH*, *HLAA*, *HPR*, *HP*, *IL1B*, *IL12B*, *IL12RB1*, *IL10*, *IL4R*, *MIF* and *TNFA* ( Bucheton *et al.*, 2011; Courtin *et al.*, 2006; Genovese *et al.*, 2010; Hardwick *et al.*, 2013; Ilboudo *et al.*, 2012; Ilboudo *et al.*, 2014; Jamonneau *et al.*, 2012; Kato *et al.*, 2015; MacLean *et al.*, 2004; Sternberg *et al.*, 2005; Stijlemans *et al.*, 2014).

Second, genome sequence data from 230 residents of HAT endemic regions in DRC, Guinea Conakry, Ivory Coast and Uganda (TrypanoGEN consortium, sequences at European Nucleotide Archive Study: EGAS00001002602) and 1000 Genomes Project data ( 1000 Genomes Project Consortium *et al.*, 2012) from African populations were used to identify sets of unlinked SNP (r ^2^<0.5) across *IL6, IL4, IL8, IFNG,* and *HLA-G* ( Courtin *et al.*, 2007; Courtin *et al.*, 2013; Ilboudo *et al.*, 2014; Lejon *et al.*, 2002; Sternberg *et al.*, 2005) ( MacLean *et al.*, 2004) using the ‘--indep-pairwise 50 5 0.5’ command in plink Plink v1.9 to select SNP with r ^2^ < 0.5 from sliding windows of 50 SNP moving 5 SNP at a time ( Chang *et al.*, 2015). A complete list of dbSNP identifiers of SNPs selected is shown in Table S1, see *Extended data* ( Fataki Asina, 2020).

### Data and statistical analysis

The results were analyzed using Plink V1.9 software ( Chang *et al.*, 2015; Purcell *et al.*, 2007) and R ( R Core Team, 2008) for viewing. Markers with >40% missing data (--geno 0.4) were removed and individuals with >30% (--mind 0.3) missing data were removed

Power calculations were undertaken using the genetics analysis package (GAP) in R ( R Core Team, 2008; Zhao, 2007).

Fisher’s exact test was used to identify associations between phenotypes and SNP loci.

The Bonferroni correction was used to correct for multiple testing. A p value below 0.05/n was considered statistically significant, where n is the number of SNPs in a given comparison after quality control.

## Results

### Quality control of data

In the candidate gene study, 233 DNA samples were genotyped: 100 active HAT cases, 33 from subjects with latent infections and 100 negative controls with a male/female sex ratio of 0.63. Data were filtered to remove loci and individuals with excessive missing data. After examining the distribution of the data, markers with >40% missing data were removed and individuals with >30% missing data were removed. These filters removed nine SNP loci, one case, four controls and four seropositives. All remaining polymorphisms were in Hardy-Weinberg equilibrium. For the validation study, 594 DNA samples were genotyped: 164 active HAT cases and 430 negative controls with a male/female sex ratio of 0.58. There was no significant difference in sex ratio between cases and controls (p=0.56) or between discovery and validation groups (p=0.43).

### Association analysis in candidate gene study

Data for the three possible comparisons between the three phenotypes (cases versus controls; latent infections versus controls; and cases versus latent infections) were analysed separately.

***Cases and controls.*** Two SNPs in *IL4* (rs2243258 and rs2243279) and two in *IFNG* (rs2430561, rs2069718) had minor alleles that appeared to be protective against HAT in the comparison between cases and controls (odds ratios <1) before Bonferroni correction ( Table 1). After Bonferroni correction, neither remained significant (p>0.05).

**Table 1.** Analysis of association between human African trypanosomiasis patients and negative control subjects.

SNP	Gene	CHR	BP	A1	Case	Control	A2	P	OR	SE	P (HWE)
rs2243258	*IL4*	5	132012110	T	0.04	0.12	C	0.003	0.31	0.42	0.16
rs2243279	*IL4*	5	132016227	A	0.04	0.12	G	0.008	0.34	0.43	0.34
rs2430561	*IFNG*	12	68552522	A	0.09	0.20	T	0.003	0.42	0.30	1.00
rs2069718	*IFNG*	12	68550162	G	0.31	0.43	A	0.014	0.59	0.21	0.73

CHR, chromosome number; SNP, dbSNP ID; BP, physical position (base-pair in GRCh37); A1, minor allele name; Case, frequency of this allele in cases; Control, frequency of this allele in controls; A2, major allele name; P, exact p-value; OR, estimated odds ratio (for A1); SE, standard error of odds ratio; P (HWE): Hardy-Weinberg equilibrium p value for unaffected individuals.

There was no statistically significant difference in allele frequency for *CFH*, *HP*, *HPR*, *IL1B*, *IL12B*, *IL12RB1*, *IL6*, *IL8*, I *L10*, *TNFA*, *HLAG*, *HLAA*, *MIF*, and *APOL1* polymorphisms.

***Latent infections and controls.*** The minor alleles of *IFNG* (rs2430561 and rs2069718) SNPs were also protective in the comparison between latent infections and controls. The minor (T) allele of *IL6* (rs2069830) was not found at all in the 29 subjects with latent infections but was found at 7% frequency in the controls ( Table 2), suggesting that it might be protective against seroconversion.

**Table 2.** Association analysis between asymptomatic latent human Africa trypanosomiasis infection subjects and negative controls.

SNP	Gene	CHR	BP	A1	Latent	Control	A2	P	OR	SE	P (HWE)
rs2069718	*IFNG*	12	68550162	G	0.24	0.43	A	0.011	0.41	0.360	1.00
rs2069728	*IFNG*	12	68547784	T	0.44	0.27	C	0.020	2.08	0.320	1.00
rs2069830	*IL6*	7	22767137	T	0.00	0.07	C	0.032	0.00	inf	0.05
rs71889624	*IL4*	5	132013430	DEL	0.00	0.06	CTGA	0.048	0.00	inf	0.03
rs35235644	*MIF*	22	24237822	C	0.02	0.10	G	0.062	0.18	1.039	0.58

CHR, chromosome number; SNP, dbSNP ID; BP, physical position (base-pair in GRCh37); A1, minor allele name; Latent, frequency of this allele in latent infections; Control, frequency of this allele in controls; A2, major allele name; P, exact p-value; OR, estimated odds ratio (for A1); SE, standard error of odds ratio; P (HWE): Hardy-Weinberg equilibrium p value for unaffected individuals; inf, infinity; DEL, deletion.

***Cases and latent infections.*** Three SNPs in *IL4* (rs2243258, rs2243279 and rs71889624) were associated with protection against progression from latent infection to case ( Table 3). Two of these were also just as strongly associated with cases and controls, despite less than a third of the number of samples of latent infections being available. This was a consequence of a higher frequency of the minor allele in latent infections than in controls. Carriers of the T allele of rs2243258 and those of the A allele of rs2243279 of *IL4* have a reduced risked of developing illness; as do carriers of the T allele of rs2069728 of *IFNG*, for asymptomatic carriers in relation to the controls.

**Table 3.** Association analysis between latent infections and cases.

SNP	Gene	CHR	BP	A1	Case	Latent	A2	P	OR	SE	P (HWE)
rs2243279	*IL4*	5	132016227	A	0.04	0.16	G	0.003	0.21	0.52	0.25
rs2243258	*IL4*	5	132012110	T	0.04	0.16	C	0.003	0.22	0.52	0.27
rs2069830	*IL6*	7	22767137	T	0.12	0.00	C	0.004	NA	NA	0.33
rs2069728	*IFNG*	12	68547784	T	0.26	0.44	C	0.019	0.45	0.32	1.00
rs71889624	*IL4*	5	132013430	DEL	0.07	0.00	CTGA	0.030	NA	NA	1.00
rs35235644	*MIF*	22	24237822	C	0.11	0.02	G	0.044	6.36	1.03	1.00

CHR, chromosome; SNP: dbSNP ID; BP, physical position (base-pair in GRCh37); A1, minor allele; Case, frequency of minor allele in cases; Latent, frequency of this allele in latent infections; A2, major allele; P, exact p-value; OR, estimated odds ratio (for A1); SE, standard error of odds ratio; P (HWE), Hardy Weinberg equilibrium p value for unaffected individuals; DEL, deletion.

The complete absence of the T allele of rs2069830 of *IL6* in latent infections also suggests that this SNP is associated with risk of progression to active HAT. None of the associations with the latent infections remained significant after Bonferroni correction.

***Validation study.*** *IL4* and *IFNG* SNPs that were marginally significant in the candidate gene study were not significant in the validation study ( Table 4).

**Table 4.** Validation study.

Source	CHR	SNP	Gene (allele)	BP	A1	A2	P	OR	SE	P (HWE)	Cases	Controls
Candidate gene	7	rs1818879	IL6	22772727	A	G	0.810	1.07	0.31	0.52	99	
Validation study	7	rs1818879	IL6	22772727	A	G	0.640	0.89	0.21	0.23	164	430
Candidate gene	5	rs2243258	IL4	132012110	T	C	0.003	0.30	0.42	0.12	99	
Validation study	5	rs2243258	IL4	132012010	T	C	0.330	0.79	0.23	0.40	164	430
Candidate gene	12	rs2430561	IFNG	68552522	A	T	0.003	0.41	0.30	0.59	99	
Validation study	12	rs2430561	IFNG	68552422	A	T	0.810	0.94	0.18	0.70	164	430
Validation study	22	rs71785313	APOL1 (G2)	36662046	2	1	0.010	0.55	0.24	0.63	164	430
Candidate gene	22	rs71785313	APOL1 (G2)	36662046	T	A	0.480	0.75	0.40	0.45	99	96
Candidate gene	22	rs73885319	APOL1 (G1)	36661906	G	A	0.330	1.27	0.26	1.00	99	96
Validation study	22	rs73885319	APOL1 (G1)	36661806	G	A	0.330	0.82	0.19	0.71	164	430

CHR, chromosome; SNP, dbSNP ID; Gene (allele), gene and also allele for APOL1; BP, physical position (base-pair in GRCh37); A1, minor allele; A2, major allele; P, exact p-value; OR, estimated odds ratio (for A1); SE, standard error of odds ratio; P (HWE), Hardy Weinberg equilibrium p value for unaffected individuals.

## Discussion

In the candidate gene study, 87 SNPs in 17 candidate genes remained after quality control. We found suggestive associations with HAT at SNP loci in *IL4, IFNG, IL6* and *MIF*, but none of these remained significant after a Bonferroni correction. It was particularly notable that no association was found with *APOL1* despite the APOL1 protein being lytic to *T. brucei brucei* and some alleles being lytic to *T. b. rhodesiense* and *T.b. gambiense* in vitro ( Genovese *et al.*, 2010). Associations have been found with the *APOL1 G1* allele in comparisons with latent infections in Guinea ( Cooper *et al.*, 2017). In that study, latent infections had been followed for two years, during which time they remained serologically positive but parasitologically negative. In our study, latent infections were defined as serologically positive but parasitologically negative at a single visit. This different case definition and the small sample size of latent infections (n=33) may account for the difference in outcomes. Associations have also been found with *APOL1 G2* frequencies between cases and controls in *T.b.rhodesiense* infections in Uganda and Malawi ( Cooper *et al.*, 2017; Kamoto *et al.*, 2019), but another study in Uganda found no association with *APOL1 G2*. Although there is strong evidence for a role for *APOL1* in HAT, the role of the different alleles in disease response is much less clear and may depend on the immunological context.

### *IL4* 

The minor alleles of rs2243258 and 2243279 were suggestively associated with protection against development of HAT in the case-control comparison and with protection against progression from latent infection to HAT, with odds ratios of 0.28±0.06 in cases versus controls, indicating a potentially large effect on the outcome of infection. However, rs2243258 showed no sign of association with HAT in the validation study ( Table 4).

A similar study in the same Bandundu focus published in 2007 tested four SNPs in *IL4* and found no association with disease, but none of those SNPs were the ones with suggestive p values in the present study ( Courtin *et al.*, 2007). Another TrypanoGEN study in Ivory Coast using the same SNP set as in the present study found five *IL4* SNPs suggestively associated with risk of developing a latent infection (uncorrected p values <0.05) ( Ahouty *et al.*, 2017). None of those five loci were associated with disease in our study. If these are genuine associations, two different haplotypes with different modes of action may be regulating responses to infection in the two countries.

*IL4* is an anti-inflammatory cytokine that is more highly expressed in late stage or chronic infections in mice and may be involved in preventing an excessive inflammatory response, causing tissue damage ( Bakhiet *et al.*, 1996; Namangala *et al.*, 2009). However, the only study of *IL4* in humans with HAT, which we are aware of, found no differences in *IL4* levels in the cerebrospinal fluid of *T. rhodesiense*-HAT patients ( Maclean *et al.*, 2001).

### *IL6* 

*IL6* (rs2069830, T allele) was suggestively associated with protection against HAT. This result adds to the evidence from previous studies for a role for *IL6* in protection against *Tbg* HAT ( Bucheton *et al.*, 2011; Sternberg *et al.*, 2005).

A previous study in the same province of Bandundu found a significant association at *IL6*rs2069849 ( Courtin *et al.*, 2007). This SNP was not included in the present study, but this finding supports a role for *IL6* variants in the development of HAT. A recent study in Guinea found that *IL6*rs1818879 was associated with a lower risk of progressing from latent infection to active disease ( Kabore *et al.*, 2017) and an Ivorian study found that rs62449495 and rs2069830 have a protective effect against developing active *Tbg* HAT ( Ahouty *et al.*, 2017). The minor allele of this last SNP (rs2069830) was also found in the same study to be protective against progressing from latent infection to cases ( Ahouty *et al.*, 2017).

*IL6* is an inflammatory protein that plays an important role in host immunity, particularly during the acute phase of the infection. Its production increases considerably in the late phase of infection and decreases dramatically after treatment, suggesting that it plays an important role in humans infected with *Tbg* ( Lejon *et al.*, 2002).

### *IFNG* 

The minor alleles of rs2069718, rs2430561 and rs2069728 were associated with protection against progression in *Tbg* HAT in the case versus control, latent infection versus control and latent infection versus case comparisons, respectively, although the results were not significant after Bonferroni correction. Individuals with the T allele of rs2069728 had about half the risk of developing HAT in the latent infection versus case comparison, as compared to carriers of the C allele. Two of the three SNPs within the same gene were tested in an Ivorian population and there was no protective effect in a comparison of cases versus controls (p>0.05) ( Ahouty *et al.*, 2017). A previous study of cases and controls in the same focus in Bandundu ( Courtin *et al.*, 2007) revealed no association with HAT at four *IFNG* SNPs but did not include those that were suggestively associated in this study. SNP rs2430561 was included in the validation study and no evidence of any association was found ( Table 4). A parallel study in Guinea with the same SNP (rs2430561) as the present study also found that *IFNG* was not associated with HAT in both case versus latent infection and latent infection versus control comparisons ( Kabore *et al.*, 2017). Recent studies on humans suggest that *IFNG* can be associated with neurological symptoms in *Tbr* HAT ( Kato *et al.*, 2016) but it is not known if this is also true of *Tbg* HAT. *IFNG* is involved in parasite control in mice ( Hertz *et al.*, 1998) and recently, its production was found to be elevated above background in the plasma of individuals with latent infections after stimulation with *Tbg* lysates but not in controls or cases of active HAT ( Ilboudo *et al.*, 2016).

### Latent infections

It is important to note that more suggestive associations were observed in comparisons of latent infections with cases or controls than were observed in the case versus control comparison, despite the small number of latent infection samples available (only 29 after quality control). This may be the most important observation of this study and suggests that latent infections are a genetically distinct group, as has been found in Guinea and Ivory Coast ( Ilboudo *et al.*, 2016; Kabore *et al.*, 2017). Latent infections might be an important reservoir of infection, but their role and significance in the epidemiology of HAT is poorly understood. If people with latent infections have distinct genetic characteristics, it may be possible to develop genetic approaches to estimating the size of this group.

### Validation study

Despite more samples being available in the validation study, including 164 cases and 430 controls, *IL4* and *IFNG* SNPs were not significantly associated with HAT. Random sample variation may account for the difference in outcome of the candidate gene and validation studies that used two different sets of samples. The negative result of the validation study shows how important it is to validate observations from candidate gene studies using fresh datasets.

## Conclusions

In conclusion, our survey reveals that two SNPs in each of *IL4* (rs2243258, T allele, and rs2243279, A allele) and *IFNG* (rs2430561, A allele, and rs2069718, G allele) appeared to be associated with a low risk of developing symptomatic *Tbg* infection in the Congolese population; however, these associations were not significant after Bonferroni correction or validation. The genetic factors of susceptibility to infection by *Tbg* in DRC were not replicated in other populations, suggesting that genetic risk factors may vary according to the population.

## Data availability

### Underlying data

Raw genotyping data on European Genome-phenome Archive, Accession number EGAS00001004365: https://identifiers.org/ega.study:EGAS00001004365.

Access to the data will be granted to life science researchers for research purposes only. Users should write to Prof. Dieudonné Mumba Ngoyi ( mumbadieudonne@yahoo.fr) who will authorise the EGA to release the data to the named researchers after completion of a data access agreement.

Harvard Dataverse: SNPs in IL4 and IFNG show no protective associations with human African trypanosomiasis in the Democratic Republic of the Congo: a case-control study. https://doi.org/10.7910/DVN/I7ODO6 ( Fataki Asina, 2020).

This project contains the following underlying data:

- DRC_CAS_CON_Candidate_Validation.xls (complete Plink output for the four SNP for used in the validation study)
- DRC_Revised_Candidate_Gene_Analysis-1.xlsx (Complete Plink output for all SNP for the four contrasts: 1) Cases v Controls 2) Latent (SERO) v Cases 3) Latent (SERO) v Controls 4) Latent (SERO) and Cases v Controls)

Data are available under the terms of the Creative Commons Zero "No rights reserved" data waiver (CC0 1.0 Public domain dedication).

### Extended data

Harvard Dataverse: SNPs in IL4 and IFNG show no protective associations with human African trypanosomiasis in the Democratic Republic of the Congo: a case-control study. https://doi.org/10.7910/DVN/I7ODO6 ( Fataki Asina, 2020).

This project contains the following extended data:

- SupplementaryDataTable_S1.txt (Table S1, SNPs genotyped in this study including rs id and co-ordinates in GRCh37 genome build)

Data are available under the terms of the Creative Commons Zero "No rights reserved" data waiver (CC0 1.0 Public domain dedication).
