# Supplementary material for: SNPs in IL4 and IFNG show no protective associations with human African trypanosomiasis in the Democratic Republic of the Congo: a case-control study
Source: AAS Open Res. 2020 Aug 4;3:35. [Version 1] doi: 10.12688/aasopenres.12999.1 (PMC7481849; doi:10.12688/aasopenres.12999.1)
Supplement: Peer review history

# Peer review: Reviewer response for version 1

**DOI:** 10.21956/aasopenres.14084.r27777

**Published:** 2020-09-09

**Authors:** Charles D. Kato

**Affiliations:** 1 School of Bio-security, Biotechnical & Laboratory Sciences, College of Veterinary Medicine, Animal Resources & Bio-security, Makarere University, Kampala, Uganda

Human African trypanosomiasis especially gHAT has been shown to present with a varying clinical outcome and disease course. Unlike rHAT that is acute and fatal, gHAT has been characterized with spontaneous cure among patients refusing treatment. The factors responsible for this varied response and outcome are still interesting areas for research. In this manuscript, the authors look at host genetic variation as one of the key factors. The authors have investigated polymorphisms within 17 candidate genes ( *APOL1 *[ *G1 and G2*], *CFH, HLA-A, HPR, HP, IL1B, IL12B, IL12RB1, IL10, IL4R, MIF, TNFA, IL6, IL4, IL8, IFNG*, and *HLA-G*). This research is of interest and will contribute towards our understanding of gHAT clinical spectrum and thus guide the design of sustainable control approaches. However, a number of improvements as highlighted below are required so as to make the work more interesting and conclusive.

- Throughout the write out, including the conclusion, the authors should clearly indicate that the results are for gHAT since these findings might not directly apply to rHAT that was not considered in this manuscript.
- I would have liked to see improvements in the background section. The study concludes as “no evidence of an association of HAT with *IL4 *and *IFNG *SNPs or with *APOL1 G1 *and *G2 *alleles, which have been found to be protective in other studies”.  However, the introduction elaborates more about other markers and does not give evidence of studies that have reported protective infects of IL4 and IFNg.
- Within the Introduction section, the authors need to provide references for this statement “It is now recognized that some individuals self-cure after developing a latent infection”.
- “For the validation study, 594 DNA samples were genotyped”. Validation of a marker is a critical step in association studies. However, the connection between the discovery and validation steps are not well articulated. The authors say they validated with 594 samples but it is not clear about which population and sampling period that these samples were collected. Were these samples collected from the same locality and matched accordingly? My other concern is why the validation step did not include latent infection. If this was an issue of getting samples then it should be indicated.
- The last sentence in the result section of the abstract “A validation study using more samples was run to determine if the absence of significant association was due to lack of power” is hanging and appears incomplete.
- About the genetic analysis I would have liked to see the different polymorphisms within the analysed gene and how these loci are linked.

Is the work clearly and accurately presented and does it cite the current literature?

Yes

If applicable, is the statistical analysis and its interpretation appropriate?

Yes

Are all the source data underlying the results available to ensure full reproducibility?

Yes

Is the study design appropriate and is the work technically sound?

Yes

Are the conclusions drawn adequately supported by the results?

Yes

Are sufficient details of methods and analysis provided to allow replication by others?

Partly

Reviewer Expertise:

Molecular biology, Immunology and Microbiology

I confirm that I have read this submission and believe that I have an appropriate level of expertise to confirm that it is of an acceptable scientific standard.

# Peer review: Reviewer response for version 1

**DOI:** 10.21956/aasopenres.14084.r27697

**Published:** 2020-08-18

**Authors:** Emile Chimusa

**Affiliations:** 1 Department of Pathology, Division of Human Genetics, University of Cape Town, Cape Town, South Africa

The manuscript by Fataki *et al.* presents a study that examines genetic variation in different outcomes of Infection with trypanosomes. They leverage a case-control design study, a total of 233 samples were recruited in the district of Masi-Manimba, in the province of Kwilu from Democratic Republic of the Congo, and were genotyped of which 100 active HAT cases, 33 from subjects with latent infections and 100 negative controls. The study by Fataki *et al.* is relevant in the field, it is clear, well written and timely, particularly the DRC data is important. However, I have major concerns on the statistics/data analysis.

1. It is not clear how the validation analysis was conducted using the additional 594 individuals, including 164 cases and 430 controls. The authors need to elaborate and make it more clear.
2. The study presented by Fataki *et al.* suffers from lower sample size, it could be much better to increase power by performing meta-analysis of DRC against the additional 594 individuals, including 164 cases and 430 controls.
3. The study by Fataki *et al.* can benefit from performing the distribution of minor allele frequency among case-control DRC and compare with the additional 594 individuals, including 164 cases and 430 controls and also 1000 Genomes Project data and importantly discussion on population specific minor allele frequency at these genotyped polymorphisms.
4. It could be much better to provide heatmap Linkage disequilibrium (LD) at the genotyped gene regions and discuss patterns of LD among these sample settings.  

Is the work clearly and accurately presented and does it cite the current literature?

Yes

If applicable, is the statistical analysis and its interpretation appropriate?

Yes

Are all the source data underlying the results available to ensure full reproducibility?

Yes

Is the study design appropriate and is the work technically sound?

Yes

Are the conclusions drawn adequately supported by the results?

Yes

Are sufficient details of methods and analysis provided to allow replication by others?

Partly

Reviewer Expertise:

Bioinformatics and Computational Biology, Population genetics and Genome-Wide Association Studies

I confirm that I have read this submission and believe that I have an appropriate level of expertise to confirm that it is of an acceptable scientific standard.
